# An emergency ECG sign of ST elevation myocardial infarction

**DOI:** 10.1186/s12872-019-1109-0

**Published:** 2019-05-30

**Authors:** Baoze Qu, Guizhou Tao, Renguang Liu

**Affiliations:** The Cardiovascular Institute of the First Affiliated Hospital of Jinzhou Medical University, Renmin Street, Jinzhou, 121000 Liaoning Province China

**Keywords:** Myocardial infarction, Acute coronary syndrome, Left anterior descending coronary artery, Electrocardiogram

## Abstract

**Background:**

The occlusion of the left anterior descending coronary artery (LAD) is usually characterized by the ST-segment elevation associated with a tall and peaked T wave in precordial leads.

**Case presentation:**

We reported a case who suffered from typical chest pain and tall and positively symmetrical T waves in leads V_2–6_, J point depression with upsloping ST-segment depression. However, the coronary angiogram demonstrated a 100% occlusion of midshaft LAD artery.

**Conclusions:**

Recognition of this atypical electrocardiogram (ECG) pattern can ensure immediate reperfusion therapy regarding acute myocardial infarction.

## Background

For the sake of immediate treatment strategies, such as reperfusion therapy, it is usual practice to designate myocardial infarction (MI) in patients with chest discomfort, or other ischemic symptoms that develop ST elevation in two contiguous leads, as an ‘ST elevation MI’. In contrast, patients without ST elevation at presentation are usually designated as having a ‘non-ST elevation MI’. We report a case of a 100% occlusion of midshaft left anterior descending (LAD) artery demonstrated by a coronary angiogram. However, tall and positively symmetrical T waves in leads V_2_-V_6_ with J point depression without ST-segment elevation.

## Case presentation

**Fig. 1:**
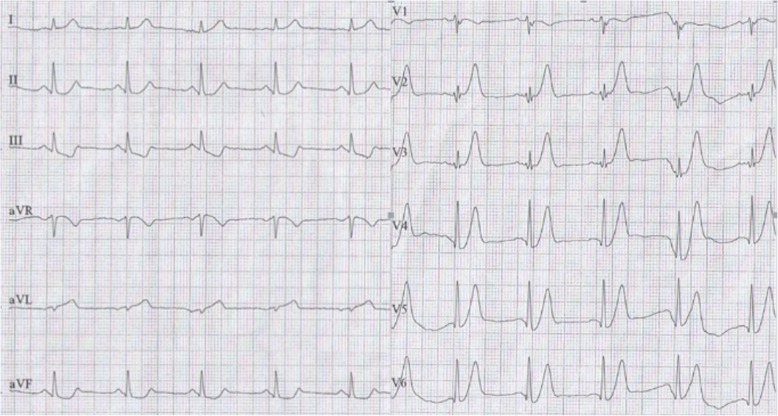
The ECG was obtained in the emergency room

**Fig. 2:**
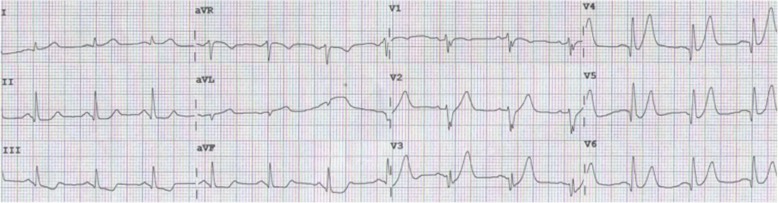
The ECG was obtained 1.5 h later, the chest pain relieved

**Fig. 3:**
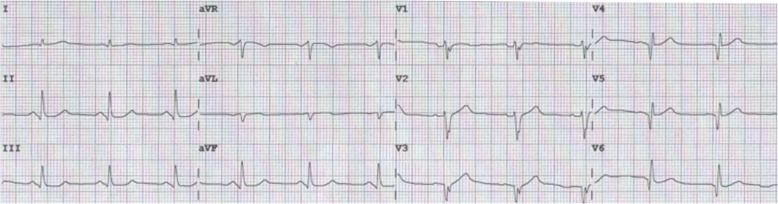
The ECG recorded 5 h after admission

**Fig. 4:**
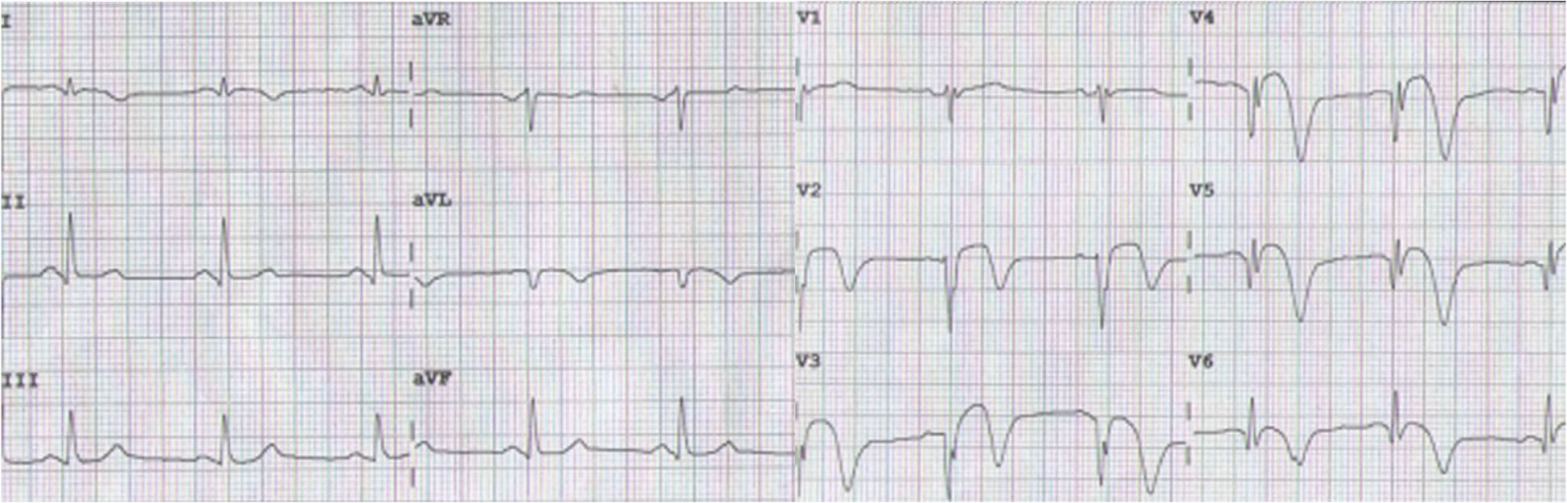
The ECG was obtained the next day after admission

**Fig. 5:**
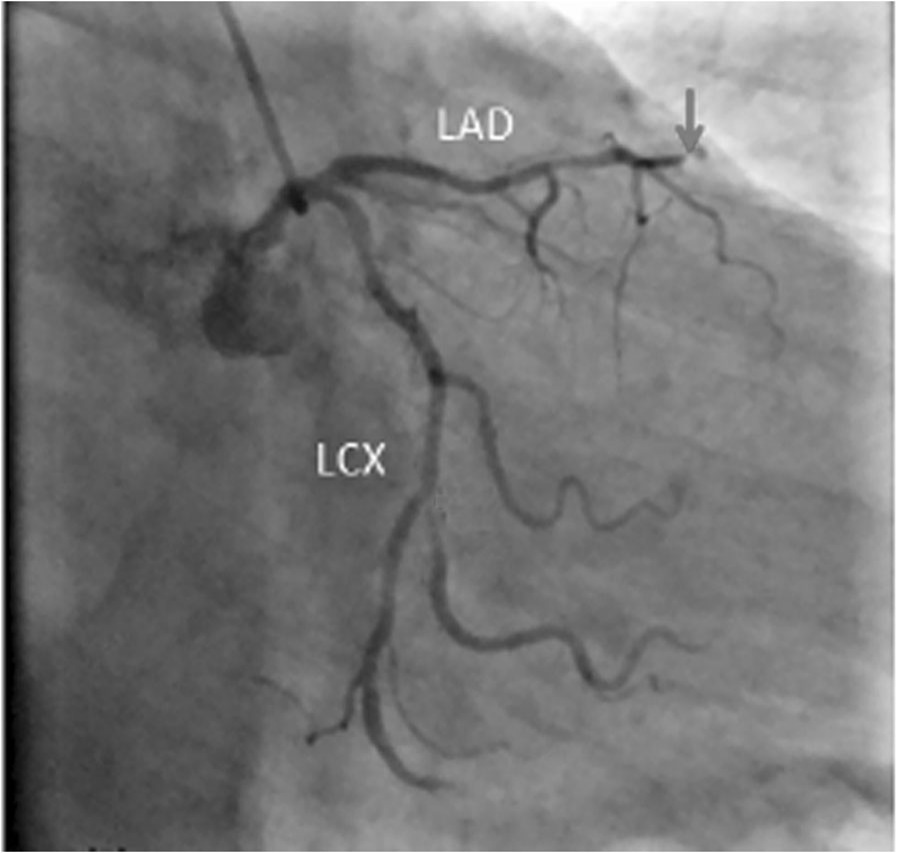
A coronary angiogram demonstrated a 100% occlusion of midshaft LAD artery

**Fig. 6:**
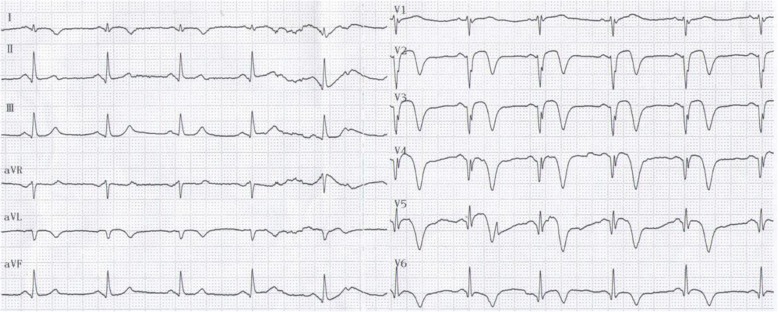
The ECG recorded the next day after coronary angiography

A 58-year-old man presented to the emergency room with 5 h of chest pain, which now has been aggravated (profuse sweating) and persistent for 0.5 h. An ECG (Fig. 1) was obtained in the emergency room which showed a sinus rhythm at a rate of 64 bpm, tall and positively symmetrical T waves in leads V_2–6_, J point depression in leads V_4–6_ (2- to 3-mm) with upsloping ST-segment depression and in leads II, III, aVF with ST-segment depression 1-mm, suggesting acute myocardial ischemia. Troponin-I was increased, which was suggestive of acute extensive anterior wall MI. The patient was immediately transferred to the catheterization laboratory for percutaneous coronary intervention. However, the patient refused underwent percutaneous coronary intervention. According to acute MI, oxygen inhalation, ECG monitoring and conventional drug therapies were adopted. 1.5 h later, the chest pain relieved and the ECG (Fig. 2) demonstrated the amplitude of tall and positively symmetrical T waves was slightly deceased in leads V_2–6_. There still existed J point depression in leads V_3–6_ with upsloping ST-segment depression. Obvious q waves appeared in leads V_3–5_, indicating that it has entered the acute phase MI. Then, the ECG (Fig. 3) recorded 5 h after admission showed that q waves in leads V_3–6_ increased, the T wave, the J point depression and ST segments in V_2–6_ leads reverted to normal, indicating the pseudo-improvement of ST-T change. The next day, the ECG (Fig. 4) revealed ST-segment elevation of leads V_2–6_ followed by T wave inversion, consistent with an ECG evolution from acute to subacute phase in patient with ST segment elevation MI (a large area). The patient agreed underwent coronary angiography and percutaneous coronary intervention. A coronary angiogram (Fig. 5) demonstrated a 100% occlusion of midshaft LAD artery. The patient and his family members chose drug therapy. The next day after coronary angiography, the ECG (Fig. 6) revealed the amplitude of ST-segment elevation decreased in leads V_3–5_. The patient revealed symptom-free 5 days after admission and then was discharged from the hospital.

## Discussion and conclusion

Our patient suffered from typical chest pain and the coronary angiogram demonstrated a 100% occlusion of midshaft LAD artery. It lacked the classic ST-segment elevation. However, his ECG characteristics included the following: (1) In hyperacute phase, tall and positively symmetrical T waves in leads V_2–6_ and J point depression with upsloping ST-segment depression lasted for necrosis Q wave appeared, mimicking non-ST elevation MI (Figs. 1 and 2). (2) In earlier acute phase (Fig. 3), Q wave appeared, and the T wave, the J and ST segment reverted to normal, indicating the transiently pseudo-improvement of ST-T change. (3) In the evolution from acute to subacute phase in ST segment elevation MI, the ECG revealed ST-segment elevation followed by T wave inversion (Figs. 4 and 6).

This special ECG pattern of LAD Occlusion was thoroughly described in the 2008 case series of de Winter et al., who recognized the characteristic ECG pattern in a primary percutaneous coronary intervention database [1]. A total of 30 of 1532 patients with anterior MI caused by LAD occlusions (2% of all cases) exhibited the characteristic ECG pattern. The ECG characteristics include the following [1–5]: (1) Instead of the signature ST-segment elevation, the ST segment showed a 1- to 3-mm upsloping ST-segment depression at the J point in leads V_1–6_ that continued into tall, positive symmetrical T waves. The tall symmetrical T waves was static, persisting from the time of first ECG until the preprocedural ECG was performed and angiographic evidence of an occluded LAD artery was obtained. (2) ST-segment elevation in lead aVR was present in the majority of cases. (3) These special changes disappeared after early reperfusion treatment (decreased R wave ST-segment elevation and T wave inversion may appear). Patients with this ECG pattern are younger, more commonly male, and have a higher incidence of dyslipidemia. (4) Tsutsumi et al. also reported that the specific feature of ST-segment changes in the inferolateral leads is associated with the acute RCA total occlusion, which is similar in appearance to the de Winter LAD pattern. Consistent with the established literature, our 58-year-old patient also exhibited the special ECG. However, there were two different points: (1) In most cases, the proximal segment of the LAD artery was occluded. Our case suggested complete occlusion of midshaft LAD artery. (2) In most cases, the feature of the initial ECG was upsloping ST-segment depression at the J point continuing into positive symmetrical T waves. However, in the earlier acute phase, the patient refused emergency percutaneous coronary intervention. Therefore, The ECG recorded pseudo-normalization of ST-T change, and the evolution of subsequent ST elevation and T wave inversion (consistent with the evolution characteristics of acute ST elevation MI). These confirmed the special ECG of the occlusion of the LAD artery was the special manifestations of early phase of ST elevation MI.

The pathophysiological mechanisms of the ECG pattern have not been elucidated yet. In any case, because of the impact in a patient’s prognosis, practitioners should recognize this LAD obstruction pattern as ST-segment elevation myocardial infarction equivalent and need to perform emergent reperfusion therapy.

## Data Availability

All relevant data supporting the conclusions of this article is included within the article.

## Abbreviations

ECG: Electrocardiogram
LAD: Left anterior descending
MI: Myocardial infarction
